# The CD47 signaling axis regulates the formation and vulnerability of atherosclerotic plaques: mechanism analysis and targeting strategies

**DOI:** 10.3389/fimmu.2026.1850727

**Published:** 2026-08-05

**Authors:** Liyang Bai, Baofeng Xu, Ying Chen, Dan Fu, Lijuan Wang, Di Ma

**Affiliations:** 1 Department of Neurology, The Neuroscience Center, The First Hospital of Jilin University, Jilin University, Changchun, China; 2 Department of Neuroelectrophysiology, Zhongyi Northeast International Hospital, Shenyang, China

**Keywords:** atherosclerotic plaque, CD47, macrophage, SIRP-α, TSP-1

## Abstract

CD47-centered signaling, involving the functionally distinct CD47–SIRPα and TSP-1–CD47 axes, has been increasingly implicated in the regulation of vascular inflammation, cellular clearance, and atherosclerotic plaque remodeling. This review examines these signaling pathways within two interrelated pathological dimensions: plaque formation and plaque vulnerability. During plaque formation, TSP-1–CD47 signaling may contribute to endothelial dysfunction, leukocyte recruitment, vascular smooth muscle cell phenotypic modulation, and macrophage foam cell formation. In particular, atherosclerosis-specific experimental evidence supports a TSP-1–CD47–Nox1-dependent mechanism that promotes receptor-independent native low-density lipoprotein uptake through macrophage macropinocytosis. During plaque progression, the CD47–SIRPα axis functions primarily as an inhibitory phagocytic checkpoint. Engagement of macrophage SIRPα recruits SHP-1/2-dependent inhibitory signaling, suppresses cytoskeletal rearrangement, and impairs efferocytosis of apoptotic cells and lipid-rich cellular remnants, thereby contributing to secondary necrosis, necrotic core expansion, and persistent inflammation. In contrast, proposed effects of CD47-centered signaling on macrophage polarization, autophagy, and angiogenic responses are more context-dependent and are supported largely by indirect evidence derived from oncology, ischemic injury, wound-healing, metabolic disease, or *in vitro* models. To clarify these mechanistic boundaries, we classify the available evidence according to its source and directness, distinguishing atherosclerosis-specific findings from related vascular evidence and hypothesis-generating observations. Finally, we review current CD47-targeted therapeutic strategies and their translational limitations, including anemia, thrombocytopenia, off-target phagocytosis, uncertain effects on vascular repair, and the lack of cardiovascular clinical evidence. Although plaque-targeted delivery and cell-selective modulation may improve therapeutic specificity, the efficacy and long-term safety of CD47-targeted interventions in human atherosclerotic cardiovascular disease remain unproven. Finally, we address key translational bottlenecks—including systemic hematological toxicities like anemia—and discuss how plaque-specific biomimetic nano-delivery systems represent the mandatory path forward to safely realize the therapeutic potential of CD47-targeted interventions in clinical cardiology.

## 1 Introduction

Cardiovascular diseases (CVD) and cerebrovascular diseases (CBVD), as the major fatal and disabling diseases worldwide, have witnessed a continuous increase in incidence, posing a significant threat to human health (1, 2). According to data from the World Health Organization (3), CVD and CBVD cause millions of deaths each year and are closely associated with high incidence rates and long-term disabilities. Especially in the context of an aging society, the social and economic burden of such diseases continues to increase. Epidemiological studies have shown that ischemic cardiovascular and cerebrovascular events, such as myocardial infarction and ischemic stroke, are closely associated with atherosclerosis, which represents their major pathological basis (4, 5).

Atherosclerosis is a chronic inflammatory disease characterized by lipid deposition on the inner wall of blood vessels, infiltration of inflammatory cells, and abnormal proliferation of smooth muscle cells. This process not only leads to narrowing of the vessel lumen but also increases the risk of plaque rupture and thrombosis, thereby triggering acute cardiovascular and cerebrovascular events (6). A plaque is typically composed of a lipid core, a fibrous cap, and a basal layer. The fibrous cap is mainly formed by smooth muscle cells that migrate into the vascular lumen and the extracellular matrix they secrete, playing a crucial role in the structural stability of the plaque (7). As the disease progresses, the lipid core within the plaque increases in size, the fibrous cap becomes thinner, and the local inflammatory response intensifies. Eventually, this may lead to the rupture of the fibrous cap, exposing the plaque contents to the bloodstream, triggering thrombosis and acute events (8). The mechanism of plaque formation and progression is highly complex and is the result of multiple factors interacting with each other. On the one hand, the inflammatory response plays a central role in the vulnerability of the plaque (9): neutrophils, macrophages and T cells increase the necrotic core and enhance the vulnerability of the plaque by secreting inflammatory mediators. On the other hand, lipid metabolism disorders also significantly promote the formation of foam cells and the expansion of the lipid core (10). Furthermore, the formation of new blood vessels within the plaque, the activation of matrix metalloproteinases (MMPs), and local hemodynamic factors all work together to further increase the risk of plaque rupture.

Traditional studies have mainly focused on the effects of lipid metabolism abnormalities and inflammatory factors. However, recent research has revealed that the innate immune checkpoint molecule CD47 plays a crucial role in regulating the function of immune cells and the infiltration of inflammation, providing a new perspective for understanding the molecular basis of plaque vulnerability (11). CD47 is a transmembrane glycoprotein that is widely expressed on the surface of various cells. By binding to the signal-regulating protein α (SIRPα), it transmits the “don’t eat me” signal, thereby inhibiting the phagocytic clearance effect of macrophages on apoptotic cells (efferocytosis) (12). CD47 also regulates angiogenesis, endothelial function, inflammatory cell migration and platelet activation by interacting with thrombospondin-1 (TSP-1) and other members of the SIRP family (11, 13). These studies have shown that CD47 not only participates in the occurrence and development of atherosclerosis, but also may be a key immune-metabolic molecule regulating plaque stability.

This review is organized around two interrelated dimensions of atherosclerotic plaque biology: plaque formation and plaque vulnerability. First, we summarize how CD47-centered signaling regulates endothelial activation, leukocyte recruitment, macrophage lipid handling, foam cell formation, VSMC phenotypic switching, and vascular remodeling during plaque formation. Second, we discuss how the CD47–SIRPα and TSP-1–CD47 axes contribute to defective efferocytosis, necrotic core expansion, unresolved inflammation, dysregulated angiogenesis, and microvascular instability during the development of vulnerable plaques. Finally, we evaluate upstream regulatory mechanisms and current therapeutic strategies targeting CD47 signaling, with particular attention to translational limitations, safety concerns, and the distinction between direct cardiovascular evidence and indirect evidence from oncology or metabolic disease models.

Compared with previous reviews that mainly focused on CD47 as a macrophage “don’t-eat-me” signal, efferocytosis, or immune checkpoints in atherosclerosis, this review integrates the CD47–SIRPα and TSP-1–CD47 axes within the dual framework of plaque formation and plaque vulnerability. We further distinguish atherosclerosis-specific evidence from indirect or hypothesis-generating evidence derived from oncology, metabolic disease, vascular injury, wound-healing, ischemic injury, and fibrotic models. This evidence-stratified perspective may help clarify the mechanistic boundaries, translational opportunities, and safety limitations of CD47-centered therapeutic strategies in cardiovascular disease.

## 2 The structure of CD47 and its multi-ligand system

CD47 is a highly conserved and functionally complex five-span membrane (5-TM) immunoregulatory receptor. Its extracellular domain consists of a single IgV-like fold domain. Through N-linked glycosylation modification, it further enriches its ligand-binding properties. There is a conserved short peptide ring (SWF ring) between the IgV domain and the transmembrane domain, which plays a key role in maintaining the overall conformational stability of the molecule. The transmembrane region is composed of five α-helices, and the overall stability is ensured by an internal hydrogen bond network and hydrophobic interactions (14). The key disulfide bonds within the molecule (such as the Cys33-Cys263 link between the IgV domain and the transmembrane region) are crucial for ligand binding and signal transduction. Their absence can significantly disrupt the structure and biological function (15). The intracellular tail is relatively short, but there are multiple splicing isoforms. These isoforms vary across tissues and cell types, contributing to the diverse roles of CD47 in immune regulation and cell adhesion (16). As an ancient and highly conservative transmembrane receptor, CD47 is widely distributed in the mammalian body, present on the surface of almost all nucleated cells (such as macrophages, smooth muscle cells, endothelial cells, etc.) as well as red blood cells. Its level can be significantly regulated by inflammatory, hypoxic, stressful and metabolic conditions. In this general context, studies related to atherosclerosis further reveal that CD47 exhibits specific cell enrichment characteristics in pathological plaques: In human carotid and coronary atherosclerotic plaques, CD47 is mainly expressed on macrophages and foam cells derived from monocytes, especially concentrated in the necrotic core and its surrounding areas. Its abnormal expression is closely associated with impaired apoptotic cell clearance and plaque progression (17). In addition to immune cells, non-hematopoietic plaque cells, including vascular smooth muscle cell-derived populations, may also express CD47 and acquire resistance to macrophage-mediated clearance (18). Logtenberg et al.’s research found that QPCTL (glutaminyl-peptide cyclotransferase-like protein) is a key enzyme that forms pyro-glutamate modification at the N-terminal of CD47, and this pGlu modification is crucial for the binding of CD47 to SIRPα (19). These structural features provide the basis for the “don’t eat me” signal mediated by CD47 and its downstream inhibition of phagocytosis (20). Among CD47-related interactions, the CD47–SIRPα axis is the best-characterized phagocytic checkpoint. Signal regulatory protein α (SIRPα) is mainly expressed on myeloid cells, including macrophages and dendritic cells, and transmits inhibitory phagocytic signals through the recruitment of SHP-1 and SHP-2 phosphatases. In contrast, TSP-1, also known as thrombospondin-1, is a matricellular glycoprotein that binds CD47 and regulates cell adhesion, apoptosis, proliferation, angiogenesis, endothelial function, vascular remodeling, and platelet activity. The multi-ligand system enables CD47 not only to be involved in lipid accumulation and inflammation, but also to exert an influence on vascular remodeling, platelet function, and immune cell recruitment. Functionally, CD47-centered signaling in atherosclerosis can be broadly divided into two related but distinct axes. The CD47–SIRPα axis primarily acts as a macrophage phagocytic checkpoint. By engaging SIRPα on macrophages, CD47 activates SHP-1/SHP-2-dependent inhibitory signaling, suppresses cytoskeletal rearrangement, and limits efferocytosis of apoptotic cells and cellular debris. In contrast, the TSP-1–CD47 axis mainly regulates vascular cell behavior, including endothelial NO/cGMP signaling, leukocyte adhesion, oxidative stress, angiogenesis, platelet activation, macrophage macropinocytosis, and VSMC remodeling. Although these two axes may interact through the shared receptor CD47 within plaques, they should be interpreted according to their dominant cellular context and biological function. To further clarify the strength and source of evidence supporting each mechanism, the evidence discussed in this review is stratified by source type in **Table 1**.

**Table 1:** Evidence stratification by source type for CD47-centered mechanisms in atherosclerosis and related models.

Mechanistic topic	Main axis	Evidence source	Evidence-source category	Interpretation
Defective efferocytosis and necrotic core expansion	CD47–SIRPα	Human atherosclerotic plaques; ApoE−/− atherosclerosis models	A	Direct atherosclerosis-specific evidence supporting CD47 blockade in restoring macrophage efferocytosis and reducing necrotic core formation
Macrophage lipid uptake via macropinocytosis	TSP-1–CD47–Nox1	Macrophage experiments; ApoE−/− atherosclerosis models	A	Atherosclerosis-specific and mechanistic evidence supporting receptor-independent LDL uptake and foam cell formation
VSMC phenotypic switching and clearance resistance	CD47/TSP-1–CD47	VSMC-specific Cd47 knockout atherosclerosis models; vascular injury and restenosis models	A	Atherosclerosis-specific and vascular injury evidence supporting a role of CD47 in VSMC remodeling, plaque growth, and macrophage-mediated clearance
Endothelial dysfunction and leukocyte recruitment	TSP-1–CD47	Endothelial cell experiments; inflammatory vascular models	B	Atherosclerosis-related vascular evidence supporting endothelial activation, leukocyte adhesion, and vascular homeostasis mechanisms related to plaque formation
Macrophage polarization after CD47–SIRPα blockade	CD47–SIRPα	Tumor immunology models	C	Indirect evidence; findings regarding pro-inflammatory or M1-like polarization should not be directly extrapolated to plaque macrophages
Angiogenesis and vascular repair	TSP-1–CD47	Endothelial cell, wound-healing, ischemic injury, and vascular repair models	C	Context-dependent indirect evidence; direct evidence for intraplaque neovascularization remains limited
Systemic metabolic regulation	CD47–SIRPα/TSP-1–CD47	Obesity, diabetes, and MASLD/NASH models	C	Indirect systemic modifier evidence rather than direct plaque-specific mechanism
Magrolimab and other anti-CD47 agents	CD47 blockade	Oncology clinical trials and clinical development experience	D	Translational safety evidence relevant to anemia, thrombocytopenia, and off-target phagocytosis, but efficacy conclusions cannot be directly transferred to atherosclerosis

Evidence categories were defined according to the source and directness of evidence. Category A indicates atherosclerosis-specific evidence derived from human atherosclerotic plaques or experimental atherosclerosis models, such as mouse models. Category B indicates atherosclerosis-related vascular evidence derived from macrophage, endothelial cell, vascular smooth muscle cell, vascular injury, restenosis, or large-animal vascular models. Category C indicates indirect or hypothesis-generating mechanistic evidence derived from oncology, metabolic disease, wound-healing, ischemic injury, or fibrotic models. Category D indicates clinical translational safety evidence mainly derived from oncology clinical trials or clinical development experience. Indirect evidence (Categories C and D) should be interpreted cautiously and should not be considered direct proof of plaque-specific mechanisms.

## 3 CD47-centered signaling in plaque formation

### 3.1 Endothelial dysfunction and leukocyte recruitment

The TSP-1/CD47 signal can directly inhibit the activity of endothelial nitric oxide synthase (eNOS), weaken the NO/cGMP vasodilation pathway, promote local vasoconstriction and oxidative stress, thereby enhancing the inflammatory microenvironment (21, 22). Furthermore, when TSP-1 binds to CD47, it can activate the Src family kinases, thereby initiating the NF-κB signaling pathway, leading to the upregulation of adhesion molecules such as VCAM-1, ICAM-1, and E-selectin, and significantly enhancing the adhesion ability of monocytes to the arterial endothelial surface. This TSP-1/CD47/NF-κB signaling axis is considered an important mechanism for the early plaque formation in atherosclerosis (23).

In terms of transendothelial migration, CD47 is also a regulatory node for leukocyte recruitment. CD47 plays multiple roles in the regulation of endothelial cell functions, including participating in cell adhesion and transendothelial migration (TEM), as well as regulating vascular homeostasis and inflammatory responses. Studies have shown that the CD47 on the surface of endothelial cells can mediate its binding to its ligand TSP-1 or its paired receptor SIRPα/γ, thereby triggering the “outside-in” signaling pathway. Azcutia et al. demonstrated that in the inflammatory model, the endothelial cells of mice with CD47 knockout exhibited a significant decrease in the ability to recruit T cells and neutrophils; *in vitro* experiments showed that anti-CD47 inhibition could reduce the tyrosine phosphorylation of VE-cadherin and the remodeling of the cytoskeleton, indicating that CD47 plays a crucial regulatory role in maintaining the integrity of endothelial junctions and transendothelial migration (24). Soriano-Romani et al. (25) discovered that in the endothelial inflammation model induced by IL-17, the TSP-1/CD47 binding peptide could significantly reduce the expression of the endothelial cell surface adhesion molecule VCAM-1 and decrease the leukocyte-endothelial adhesion reaction, revealing that the CD47 signal can alleviate endothelial activation under inflammatory conditions.

### 3.2 Macrophage lipid handling and foam cell formation

Foam cell formation results from excessive lipid uptake together with defective clearance of apoptotic foam cells and lipid-rich cellular debris. Crucially, persistent CD47–SIRPα-mediated “don’t-eat-me” signaling does not directly constitute a lipid metabolic or transporter pathway; rather, it indirectly increases the plaque lipid burden by impairing macrophage efferocytosis of apoptotic foam cells and lipid-rich remnants. Consistent with this concept, Xia et al. (26) developed CD47p-GQDs-miR223-engineered monocytes to combine CD47-related competitive blockade with miR-223-mediated anti-inflammatory regulation. This strategy reduced macrophage ox-LDL uptake and decreased the secretion of TNF-α, IL-1β, and IL-6, thereby limiting foam cell formation and improving plaque stability in the experimental model. Distinct from the CD47–SIRPα–efferocytosis mechanism, the TSP-1–CD47 axis can more directly regulate macrophage lipid uptake by promoting receptor-independent macropinocytosis. Mechanistically, binding of thrombospondin-1 (TSP-1) to macrophage CD47 activates NADPH oxidase 1 (Nox1), induces membrane ruffling, and enhances fluid-phase macropinocytosis, thereby promoting receptor-independent internalization of native LDL and macrophage lipid accumulation. Csányi et al. (27) validated the TSP-1–CD47–Nox1–cofilin pathway as a driver of fluid-phase macropinocytosis in macrophages both *in vitro* and *in vivo*. They further showed that this pathway operates in ApoE−/− atherosclerotic mice, where it promotes macrophage foam cell formation and plaque lipid accumulation. Importantly, this process is independent of classical scavenger receptors such as SR-A and CD36, indicating that TSP-1–CD47 signaling can facilitate foam cell formation through a redox-dependent, receptor-independent LDL uptake pathway.

Additional studies further support fluid-phase macropinocytosis as an important contributor to foam cell formation within the arterial wall. Lin et al. (28) used myeloid-specific inhibition of macropinocytosis and pharmacological interventions to examine the role of receptor-independent LDL uptake in atherosclerosis. Suppression of macropinocytosis reduced arterial lipid deposition and plaque burden, whereas enhancement of this pathway increased uptake of both native and modified LDL and accelerated foam cell formation. Miyazaki (29) further summarized the role of receptor-independent endocytic pathways in macrophage foam cell formation. This review highlighted that signaling pathways such as TSP-1–CD47–Nox1 can drive membrane ruffling and macropinocytosis, thereby supplementing, and in some contexts partially compensating for, classical scavenger receptor-mediated pathways such as SR-A and CD36. This mechanism may amplify macrophage lipid uptake under conditions of high extracellular LDL burden (29). In addition, TSP-1–CD47 signaling is linked to dysregulated macrophage lipid metabolism through Nox-dependent redox pathways. Through this mechanism, TSP-1–CD47 signaling may couple lipid accumulation with chronic vascular inflammation and plaque progression (30).

The evidence supporting the TSP-1–CD47–Nox1–macropinocytosis pathway is largely atherosclerosis-related, because it has been validated in macrophage experiments and ApoE−/− atherosclerotic mouse models. Therefore, TSP-1–CD47–Nox1-mediated macrophage macropinocytosis represents an important receptor-independent pathway for LDL uptake and foam cell formation. This pathway provides a mechanistic basis for understanding how CD47-centered signaling contributes to macrophage lipid burden and early plaque lipid accumulation.

### 3.3 VSMC phenotypic switching and plaque growth

The TSP-1/CD47 axis contributes to atherosclerotic plaque development through dual mechanisms. On one hand, it promotes abnormal expansion of plaque cellular components by regulating VSMC phenotypic switching, migration, and proliferation. On the other hand, by enhancing the “don’t-eat-me” signal on VSMC surfaces, it inhibits their clearance by macrophages, indirectly enlarging the necrotic core and weakening fibrous cap stability. Recent lineage-tracing and single-cell transcriptomic studies have shown that VSMCs contribute substantially to the cellular composition of atherosclerotic plaques through phenotypic switching, and the transition from a contractile to a synthetic phenotype represents a critical determinant of plaque progression and stability. Recent studies using VSMC-specific Cd47 knockout mouse models have further confirmed the critical role of CD47 in this process at the genetic level. Pervaiz et al. generated VSMC-specific Cd47-deficient mice on an ApoE^-/- background and found that, compared with controls, these mice exhibited significantly reduced plaque area, necrotic core size, and macrophage accumulation within plaques. These findings indicate that CD47 expression on VSMCs promotes plaque burden and influences plaque stability (18). In addition to genetic evidence, cellular-level studies also support a role for TSP-1/CD47 in regulating VSMC phenotypes. TSP-1 is markedly upregulated in diseased vascular walls and in VSMCs, and exogenous TSP-1 stimulation suppresses contractile phenotype genes in VSMCs (such as ACTA2, CNN1, and TAGLN) while enhancing proliferation and migration. Knockdown of CD47 significantly attenuates these effects, indicating that the TSP-1/CD47 axis directly drives the transition of VSMCs from a contractile to a pathological “modulated” phenotype (31). Moreover, VSMCs significantly upregulate CD47 in response to vascular injury and inflammatory stimuli. In a study by Govatati et al., thrombin was shown to promote CD47 expression in VSMCs via the PAR1–Gα_q/11–PLCβ3–NFATc1 signaling pathway, enhancing their migration and proliferation. Application of CD47 siRNA or blocking antibodies not only inhibited VSMC activation but also significantly improved macrophage efferocytosis of apoptotic VSMCs, thereby reducing neointimal formation in the affected regions (32). VSMC-derived CD47 signaling may also influence plaque stability by affecting fibrous cap integrity and macrophage-mediated clearance.

### 3.4 Vascular tone and mechanotransduction

In addition to endothelial activation and macrophage lipid handling, TSP-1–CD47 signaling may also contribute to plaque formation by regulating vascular tone and endothelial mechanotransduction. The TSP-1–CD47 axis suppresses endothelial NO/cGMP signaling, thereby reducing vasodilatory capacity and increasing vascular resistance. Evidence from pulmonary hypertension models further suggests that TSP-1–CD47 activation can disrupt CD47–caveolin-1 interactions and shift eNOS activity toward superoxide rather than NO production, supporting its broader role in vascular dysfunction (33). Although this evidence is not derived from atherosclerotic plaques, it supports the broader role of TSP-1–CD47 signaling in vascular dysfunction (34). The TSP-1–CD47 axis also serves as an effector of endothelial mechanosensing, including responses to shear stress. Under *in vitro* mechanical stimulation, TSP-1–CD47-mediated signaling can trigger mechanosensitive apoptosis in endothelial cells, suggesting that this pathway translates mechanical stimuli into changes in endothelial cell fate and function (35). In addition, TSP-1–CD47 signaling suppresses shear stress-induced NO production, thereby attenuating physiological vasodilatory responses and local blood flow regulation (36). Together, these findings suggest that TSP-1–CD47 signaling regulates endothelial function not only under resting conditions but also under hemodynamic stimulation, thereby contributing to disturbed vascular homeostasis at atheroprone sites.

## 4 CD47-centered signaling in plaque vulnerability

### 4.1 Impaired efferocytosis and necrotic core expansion

Defective efferocytosis is a central pathological mechanism linking CD47–SIRPα signaling to plaque vulnerability, as persistent activation of this phagocytic checkpoint suppresses macrophage-mediated clearance of apoptotic cells and lipid-rich debris, thereby leading to secondary necrosis, necrotic core expansion, inflammatory amplification, and reduced fibrous cap stability (37). This mechanism may be amplified in lipid-rich and inflamed plaques, where oxidative stress and metabolic stress enhance inhibitory phagocytic signaling and reduce the efficiency of apoptotic cell clearance. Among the mechanisms discussed in this review, the role of CD47–SIRPα signaling in defective efferocytosis is supported by the most direct atherosclerosis-specific evidence, including human plaque observations and experimental atherosclerosis models.

At the molecular level, CD47 on apoptotic cells, foam cells, or other plaque-associated cells binds SIRPα on macrophages and activates inhibitory signaling through the cytoplasmic immunoreceptor tyrosine-based inhibitory motifs of SIRPα. This interaction induces phosphorylation of SIRPα immunoreceptor tyrosine-based inhibitory motifs and recruits SHP-1 and SHP-2 phosphatases. As a result, downstream pro-phagocytic signaling, actin cytoskeletal rearrangement, and engulfment of apoptotic cells are suppressed, resulting in impaired macrophage efferocytosis. In atherosclerosis, this inhibitory pathway may counteract pro-efferocytic signals such as low-density lipoprotein receptor-related protein 1 (LRP1), thereby weakening macrophage clearance of apoptotic cells and lipid-rich debris (17). Sustained “don’t-eat-me” signaling therefore promotes the retention of dead cell debris and lipid-rich remnants, creating a pro-inflammatory microenvironment within the plaque. Conversely, blockade of CD47–SIRPα signaling can restore macrophage efferocytosis and reduce the accumulation of apoptotic cells, lipid-rich debris, and necrotic material within plaques. Elevated CD47 expression on plaque-associated apoptotic cells, foam cells, and VSMC-derived cells can inhibit their macrophage-mediated clearance, resulting in apoptotic cell accumulation, necrotic core enlargement, and persistent inflammatory activation (38). Uncleared apoptotic cells subsequently undergo secondary necrosis, releasing lipids, damage-associated signals, and inflammatory mediators that amplify local inflammation within plaques (39). Importantly, studies in atherosclerosis models have shown that CD47 blockade enhances macrophage efferocytosis, reduces necrotic core size, and improves features of plaque stability, supporting CD47 as an important regulatory node in defective plaque efferocytosis (11).

Beyond defective efferocytosis, autophagy dysfunction may further modulate CD47-centered inflammatory responses in plaques. Autophagy is involved in endothelial homeostasis, lipid handling, oxidative stress control, and inflammatory regulation during atherosclerosis. Experimental studies suggest that impaired autophagic flux can promote endothelial dysfunction and inflammatory activation through pathways such as PI3K/AKT/mTOR and eNOS signaling (40–42). In addition, CD47 itself may be regulated by ubiquitination and autophagy-dependent degradation; impaired degradation may increase cell-surface CD47 and reinforce “don’t-eat-me” signaling, whereas autophagy induction may reduce CD47 accumulation in some immunotherapy models (43). However, because much of this evidence is indirect or derived from non-atherosclerotic models, autophagy should be discussed as a potential modifier rather than as a fully established CD47–SIRPα mechanism in plaque vulnerability.

In summary, the CD47–SIRPα axis promotes plaque vulnerability primarily by suppressing macrophage efferocytosis. This leads to apoptotic cell retention, secondary necrosis, necrotic core expansion, and persistent inflammatory activation. Autophagy dysfunction and endothelial injury may further amplify this process, but these mechanisms should be interpreted as complementary modifiers rather than as substitutes for the central efferocytosis pathway.

### 4.2 Macrophage phenotype, efferocytosis, and inflammation resolution

The relationship between CD47–SIRPα signaling, macrophage phenotype, inflammation, and efferocytosis is highly context-dependent. In atherosclerotic plaques, the most consistently supported role of this axis is the inhibition of macrophage efferocytosis. Blockade of CD47–SIRPα signaling releases this inhibitory checkpoint and enhances macrophage-mediated clearance of apoptotic cells, foam cells, and lipid-rich debris. However, the accompanying macrophage inflammatory phenotype may vary substantially across disease models and microenvironmental contexts. Therefore, evidence that CD47–SIRPα blockade promotes a pro-inflammatory or M1-like macrophage phenotype should be interpreted according to its model source. Such findings are mainly derived from tumor immunology models and should be regarded as indirect and hypothesis-generating rather than direct evidence for macrophage polarization in atherosclerotic plaques. Indirect evidence from tumor-related inflammatory models further supports the context-dependent nature of CD47–SIRPα-mediated macrophage regulation. In tumor models, Sakamoto et al. (44) showed that antibodies targeting SIRPα/SIRPβ1 promoted tumor-infiltrating macrophages toward an antitumorigenic phenotype, suggesting that interference with SIRP-related signaling can reshape macrophage functional states. Similarly, Zimarino et al. (45) reported in tumor-associated myeloid-derived suppressor cells that disruption of CD47–SIRPα signaling restored phagocytosis and antigen presentation, accompanied by renewed T-cell activity. These findings indirectly suggest that CD47–SIRPα signaling may regulate not only phagocytic activity but also broader myeloid immune functions, although the direction of inflammatory polarization may differ across disease contexts. In the oncology context, Gao et al. (46) summarized that CD47–SIRPα blockade may shift tumor-associated macrophages toward a more pro-inflammatory, antitumor phenotype, thereby reshaping the tumor immune microenvironment. However, these findings are derived from tumor immunology and should not be directly extrapolated to atherosclerotic plaques, where macrophage activation, lipid handling, efferocytosis, and inflammation resolution follow a distinct pathological logic. In atherosclerosis, the major therapeutic rationale for CD47–SIRPα blockade is to restore macrophage efferocytosis, enhance the clearance of apoptotic cells and foam cells, limit secondary necrosis and necrotic core expansion, and promote inflammation resolution. Therefore, tumor-derived evidence regarding macrophage polarization should be interpreted as indirect and hypothesis-generating, whereas conclusions about plaque inflammation should be based primarily on atherosclerosis-specific evidence whenever available.

### 4.3 Context-dependent regulation of angiogenesis and microvascular instability

The TSP-1–CD47 signaling axis regulates angiogenesis in a highly context-dependent manner within vascular and plaque microenvironments. Activation of this axis generally suppresses VEGF-dependent endothelial responses, whereas CD47 blockade or deficiency can relieve this inhibitory brake and enhance angiogenic responsiveness under hypoxic or reparative conditions.

Mechanistically, TSP-1–CD47 activation has been widely characterized as an anti-angiogenic pathway. Kaur et al. found that binding of TSP-1 to endothelial CD47 disrupts CD47–VEGFR2 signaling and markedly inhibits VEGFR2 phosphorylation as well as downstream Akt and eNOS activation (47). This suppresses endothelial cell migration and tube formation, thereby inhibiting VEGF-dependent angiogenesis. In brain microvascular endothelial cells, exogenous TSP-1 stimulation reduces VEGF-induced tube formation and migration, further supporting the cellular basis for TSP-1–CD47-mediated angiogenic inhibition. Under chronic pathological conditions, such as diabetic wounds, sustained activation or high expression of TSP-1–CD47 signaling is associated with endothelial senescence, impaired proliferation, and deficient reparative angiogenesis (48). From an oxidative stress perspective, Meijles et al. showed that TSP-1–CD47–Nox1 signaling activates pro-oxidant pathways, promotes endothelial senescence, and exacerbates vascular dysfunction (49). Within atherosclerotic plaques, excessive TSP-1–CD47 activity may reduce endothelial NO production, impair vasodilatory responses, and limit reparative endothelial function. In the plaque microenvironment, impaired endothelial repair and microvascular dysfunction may contribute to fragile neovessels, intraplaque hemorrhage, and plaque instability.

Conversely, CD47 inhibition or deficiency can enhance angiogenic responsiveness under conditions of hypoxia, acute injury, or high reparative demand. In a thermal injury model, Soto-Pantoja et al. (50) demonstrated that CD47-deficient mice showed increased perfusion and vascularization at the wound edge, together with accelerated wound closure. Pharmacological blockade of TSP-1–CD47 signaling similarly improved tissue revascularization, suggesting that inhibition of this axis can enhance reparative angiogenesis. Mechanistically, CD47 deficiency may relieve TSP-1-mediated inhibition of VEGFR2 signaling, thereby amplifying VEGF responsiveness, mitochondrial metabolism, and eNOS activity in endothelial cells (49, 51). In the context of atherosclerosis, this pro-angiogenic response should be interpreted cautiously, as direct evidence within plaques remains limited; it may be more relevant to reparative angiogenesis in ischemic or injured vascular tissues than to stable intraplaque neovascularization. Such pro-angiogenic responses are most likely to occur in microenvironments characterized by strong VEGF signaling, tissue hypoxia, injury repair, or pharmacological suppression of CD47 signaling.

Evidence regarding TSP-1–CD47-mediated angiogenic regulation is mainly derived from endothelial cell, wound-healing, ischemic injury, and vascular repair models; therefore, its relevance to intraplaque neovascularization and plaque vulnerability should be interpreted cautiously until direct plaque-specific evidence becomes available. Thus, the role of TSP-1–CD47 signaling in angiogenic regulation should be understood as context-dependent pathway modulation rather than a simple bidirectional effect. In plaque microenvironments dominated by high TSP-1 expression, inflammation, and oxidative stress, pathway activation mainly suppresses endothelial VEGF responsiveness and impairs vascular repair; whereas under hypoxia, injury repair, or CD47 blockade, release of this inhibitory signal may enhance angiogenic responsiveness. This context dependency indicates that TSP-1–CD47 signaling may influence plaque stability by regulating endothelial repair, neovascular responsiveness, oxidative stress, and microvascular integrity, but direct plaque-specific evidence remains limited and should be distinguished from injury-repair models.

### 4.4 Crosstalk between the CD47–SIRPα and TSP-1–CD47 axes

Although the CD47–SIRPα and TSP-1–CD47 axes are generally considered functionally distinct—primarily regulating phagocytic checkpoint inhibition and vascular cell function, respectively—they are not entirely independent within atherosclerotic plaques, which represent highly inflamed, cellularly complex, and signal-rich microenvironments. These pathways may interact through the shared receptor CD47, ligand competition or co-occupancy, and synergistic remodeling of macrophage, endothelial cell, and VSMC states.

As discussed above, defective efferocytosis is a central event in necrotic core formation and sustained plaque inflammation. Within mature plaques, upregulation of CD47 on apoptotic cells, foam cells, and VSMC-derived plaque cells can engage SIRPα on macrophages and inhibit pro-efferocytic signaling, thereby contributing to impaired efferocytosis (52). In parallel, TSP-1–CD47 signaling can exacerbate endothelial dysfunction and oxidative stress by inhibiting NO–cGMP signaling, while also modulating angiogenic responses and microvascular integrity. These alterations may reinforce macrophage inflammatory activation, defective efferocytosis, and VSMC maladaptive remodeling, thereby creating a functional amplification loop linking endothelial dysfunction, impaired apoptotic cell clearance, necrotic core expansion, and plaque destabilization.

**Figure 1:**
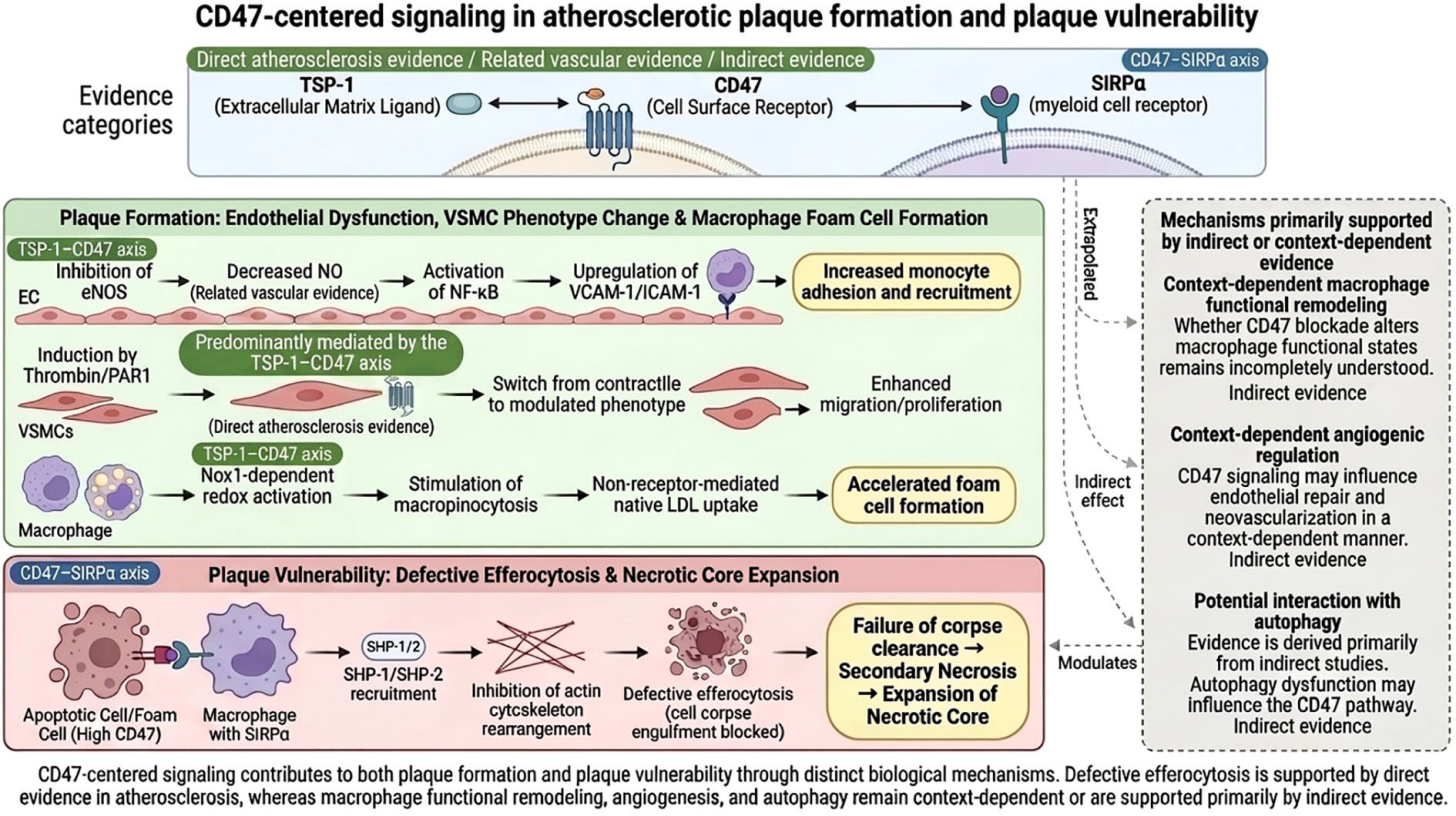
CD47-centered signaling pathways across the distinct pathological dimensions of atherosclerotic plaque formation and plaque vulnerability. This schematic delineates the biological actions of CD47-centered signaling, structurally categorized into the dual dimensions of plaque formation (top panel, green) and plaque vulnerability (bottom panel, pink), while strictly partitioning direct vascular evidence from indirect, hypothesis-generating mechanisms (right panel, gray/dashed). Under plaque formation, the TSP-1–CD47 axis mediates endothelial dysfunction (via eNOS inhibition and subsequent VCAM-1/ICAM-1 upregulation), vascular smooth muscle cell (VSMC) phenotypic switching from contractile to modulated states, and receptor-independent native LDL uptake via macrophage macropinocytosis, supported by a combination of direct atherosclerosis and related vascular evidence. Under plaque vulnerability, the CD47–SIRPα axis operates on macrophages to recruit SHP-1/2, inhibit actin cytoskeleton rearrangement, and block the engulfment of apoptotic cells, directly promoting defective efferocytosis, secondary necrosis, and necrotic core expansion (supported by direct Category A evidence). Conversely, context-dependent or indirect pathways—including macrophage functional remodeling (polarization shifts), angiogenic regulation (neovascularization), and autophagy interactions—are compartmentalized on the right and connected via dashed arrows to denote their extrapolated or indirect nature, thereby ensuring the graphical representation aligns precisely with the evidence hierarchy established in the text.

Therefore, the crosstalk between these two axes should be understood not as functional equivalence, but as convergence of immune checkpoint inhibition and vascular stress signaling on plaque vulnerability. The integrated roles of CD47–SIRPα and TSP-1–CD47 signaling in plaque formation and vulnerability are summarized in **Figure 1**.

## 5 Upstream and systemic regulation of CD47-centered signaling

The mechanisms discussed above mainly describe how CD47-centered signaling directly regulates plaque formation and vulnerability at the cellular level. In addition to these plaque-level mechanisms, CD47 signaling is also shaped by upstream post-transcriptional regulators and systemic metabolic cues. These factors do not necessarily act as direct plaque-effector mechanisms, but they may modulate the intensity, cellular distribution, and pathological consequences of CD47–SIRPα and TSP-1–CD47 signaling in atherosclerosis.

### 5.1 miRNA-mediated upstream regulation

miRNAs should be considered upstream regulatory molecules rather than direct plaque-effector mechanisms. By modulating the transcriptional or post-transcriptional expression of CD47, SIRPα, or their related signaling components, miRNAs may indirectly affect macrophage efferocytosis, inflammatory phenotype, cholesterol handling, and plaque stability. Among atherosclerosis-related studies, miR-378a provides relatively direct evidence for this regulatory framework. miR-378a has been reported to modulate macrophage phagocytosis and differentiation through the CD47–SIRPα axis, mainly by downregulating SIRPα expression rather than directly suppressing CD47. By reducing SIRPα-mediated inhibitory signaling, miR-378a may enhance macrophage efferocytosis, facilitate apoptotic-cell clearance, attenuate inflammatory activation, and alleviate plaque progression (53). This is consistent with the broader role of the CD47–SIRPα axis as a negative regulator of monocyte/macrophage phagocytic function in atherosclerosis (54).

Compared with atherosclerosis, evidence for miRNA-mediated regulation of CD47 is more abundant in tumor immunology. For example, in pancreatic ductal adenocarcinoma models, miR-128 indirectly reduced CD47 expression through ZEB1 downregulation, whereas miR-340 directly targeted the 3′UTR of CD47 and restored macrophage-mediated phagocytosis of tumor cells (55, 56). Although these findings were obtained in tumor models, they provide a mechanistic analogy suggesting that miRNA-based downregulation of CD47 may enhance macrophage clearance of pathological cells. However, such evidence should be interpreted as indirect and hypothesis-generating for atherosclerosis rather than as direct cardiovascular evidence.

miRNAs targeting SIRPα have also been reported. The miR-17/20a/106a cluster can directly target SIRPα and modulate macrophage inflammatory responses and phagocytic activity (57). In a renal fibrosis model, miR-382 was shown to bind the 3′UTR of SIRPα, reduce SIRPα expression, and reshape macrophage polarization through STAT3-related signaling (58). These studies demonstrate that SIRPα can be post-transcriptionally regulated by miRNAs, but their relevance to atherosclerotic plaques remains inferential. In plaque macrophages, analogous SIRPα-targeting miRNAs may theoretically attenuate “don’t-eat-me” signaling and enhance efferocytosis, but this possibility requires direct validation in atherosclerosis models.

Among miRNA-related mechanisms, miR-378a provides relatively plaque-relevant evidence through regulation of SIRPα-mediated macrophage phagocytosis. In contrast, evidence for miR-128, miR-340, miR-382, and the miR-17/20a/106a cluster is mainly derived from tumor, inflammatory, or fibrotic models and should be regarded as indirect mechanistic support. Taken together, miR-378a currently provides the most plaque-relevant evidence for miRNA-mediated modulation of the CD47–SIRPα axis in atherosclerosis, whereas miR-128, miR-340, miR-382, and the miR-17/20a/106a cluster mainly provide indirect mechanistic support from tumor, inflammatory, or fibrotic disease models. Future therapeutic strategies may combine CD47/SIRPα blockade with targeted delivery of specific miRNA mimics or antisense oligonucleotides to fine-tune macrophage efferocytosis and inflammatory responses within plaques. However, the delivery efficiency, cell specificity, off-target effects, and cardiovascular safety of such approaches require further investigation (59).

### 5.2 Systemic metabolic regulation as an indirect modifier

Systemic metabolic diseases do not represent plaque-specific CD47 mechanisms, but they may indirectly modify CD47-centered signaling by altering inflammatory tone, insulin resistance, lipid metabolism, oxidative stress, endothelial function, and adipose–vascular communication. Therefore, obesity, diabetes, and MASLD/NASH should be discussed as systemic modifiers of plaque formation and vulnerability rather than as mechanisms equivalent to macrophage efferocytosis or endothelial dysfunction.

Experimental studies suggest that CD47 and TSP-1–CD47 signaling participate in systemic metabolic regulation. CD47 deficiency has been associated with protection against high-fat diet-induced obesity, improved glucose tolerance and insulin sensitivity, and reduced adipose and hepatic inflammation (60). TSP-1 has also been identified as an adipokine associated with obesity, adipose inflammation, insulin resistance, and cardiometabolic risk (61). At the islet level, CD47-related signaling has been implicated in β-cell function and macrophage-mediated β-cell clearance, suggesting that this pathway may also influence glucose metabolism and systemic metabolic homeostasis (62, 63). These metabolic effects may indirectly influence atherosclerosis by reducing chronic low-grade inflammation, improving lipid handling, and alleviating endothelial stress.

In hepatic metabolic disease, CD47–SIRPα signaling has been linked to NASH/MASLD progression, hepatocyte clearance, hepatic inflammation, and fibrosis (64). Because MASLD is closely associated with insulin resistance, dyslipidemia, systemic inflammation, and endothelial dysfunction, modulation of CD47-related pathways in this context may indirectly affect atherosclerotic plaque development. However, these findings should be interpreted as systemic metabolic evidence rather than direct plaque-specific mechanisms.

These findings should be interpreted as indirect systemic modifier evidence rather than plaque-specific mechanisms, because metabolic diseases may influence atherosclerosis through systemic inflammation, insulin resistance, dyslipidemia, and endothelial stress. From a translational perspective, these findings suggest a potential link between metabolic control and CD47-centered vascular inflammation. Conventional metabolic interventions, including weight reduction, lipid-lowering therapy, glucose control, and lifestyle modification, may indirectly attenuate plaque inflammation by improving systemic metabolic homeostasis. In selected metabolic disease contexts, combined metabolic–immune strategies targeting CD47–SIRPα or TSP-1–CD47 signaling may represent a future research direction. Nevertheless, whether such approaches can directly stabilize atherosclerotic plaques remains to be established in dedicated cardiovascular models and clinical studies.

## 6 Therapeutic strategies targeting CD47 and their research progress

Based on the mechanisms discussed above, therapeutic strategies targeting CD47-centered signaling can be broadly divided into antibody- or fusion protein-based blockade, small-molecule and RNA-based modulation, and plaque-targeted nanodelivery systems. These approaches aim to restore macrophage efferocytosis, reduce necrotic core formation, attenuate inflammatory amplification, and improve plaque stability. However, because CD47 is broadly expressed on erythrocytes, platelets, endothelial cells, leukocytes, and other normal tissues, therapeutic translation requires careful consideration of hematologic toxicity, platelet-related adverse effects, off-target phagocytosis, and long-term safety. Representative CD47-centered therapeutic strategies, including antibody-based blockade, SIRPα-related interventions, small-molecule and RNA-based approaches, and nanodelivery systems, are summarized in **Table 2** with their corresponding models, target cells, outcomes, limitations, and evidence sources.

**Table 2:** Representative CD47-centered therapeutic strategies in atherosclerosis and related vascular models.

Therapeutic strategy	Representative intervention	Experimental model	Route/delivery method	Target cell type/tissue	Main outcomes	Limitations/translational concerns	Reference number
CD47–SIRPα checkpoint blockade	Anti-CD47 antibody	ApoE−/− mouse atherosclerosis models; human atherosclerotic plaque specimens	Systemic anti-CD47 antibody administration	Plaque macrophages, apoptotic cells, foam cells, and VSMC-derived plaque cells	Restored macrophage efferocytosis, reduced plaque burden, decreased necrotic core formation, and improved plaque stability	Systemic CD47 blockade may induce anemia, thrombocytopenia, off-target phagocytosis, and limited plaque selectivity	(11, 17)
CD47 blockade in vascular injury/restenosis	Anti-CD47 antibody or CD47 blockade	Mouse carotid artery injury/restenosis model	Systemic administration or CD47 blockade	VSMCs and macrophages in injured vascular wall	Promoted engulfment of apoptotic VSMCs, inhibited VSMC migration and proliferation, and reduced restenosis severity	Vascular injury and restenosis models are not equivalent to spontaneous atherosclerotic plaque vulnerability	(32)
SIRPα-related blockade or SIRPα deletion	SIRPα–Fc fusion proteins or SIRPα-related blocking agents	Cancer immunotherapy models; high-fat diet-induced atherosclerosis-related models	Soluble SIRPα–Fc fusion protein or SIRPα-related blockade	Macrophage SIRPα and CD47-expressing pathological cells	Competitively disrupted CD47–SIRPα interaction and enhanced macrophage phagocytic activity	Most clinical development remains oncology-oriented; cardiovascular evidence remains limited	(37, 74, 76)
Next-generation CD47-targeting antibodies	IMC-002; AK117/ligufalimab	Solid tumor or oncology models	Engineered anti-CD47 monoclonal antibody administration	CD47-expressing pathological cells; erythrocyte-sparing CD47 targeting	Reduced red blood cell binding, hemagglutination, and hematologic toxicity compared with first-generation anti-CD47 antibodies	Evidence is mainly derived from oncology; plaque selectivity and long-term cardiovascular safety remain unknown	(71, 72)
Downstream checkpoint modulation	SHP-1 inhibition	Atherosclerosis-related mouse models; macrophage-targeted nanocarrier models	Small-molecule SHP-1 inhibitor or nanocarrier-mediated delivery	Plaque macrophages and SHP-1-mediated inhibitory signaling	Enhanced macrophage efferocytosis, reduced inflammatory activation, and limited plaque progression	Requires efficient macrophage-targeted delivery; systemic SHP-1 modulation may cause off-target immune effects	(79, 80)
miRNA-based modulation	miR-378a mimic	ApoE−/− mouse atherosclerosis model; macrophage functional assays	RNA mimic-based intervention	Macrophage SIRPα-related signaling	Enhanced foam cell clearance, increased macrophage efferocytosis, reduced inflammatory mediator production, and attenuated plaque progression	RNA delivery efficiency, cell specificity, off-target effects, and long-term cardiovascular safety remain unresolved	(53)
Combined pro-efferocytic and anti-inflammatory nanotherapy	SHP-1 inhibitor plus IL-10 co-delivery	Murine atherosclerosis models	Macrophage-targeted polymeric nanoparticles carrying NSC87877 and IL-10	Plaque macrophages	Simultaneously enhanced phagocytosis and promoted inflammation resolution, reducing plaque burden and inflammatory activation	Preclinical evidence only; nanoparticle manufacturing and long-term safety require further validation	(80)
Anti-CD47 antibody-modified nanocarrier	CS–HA nanoparticles carrying anti-CD47 antibodies	ApoE−/− mouse atherosclerosis model; vascular endothelial cell assays	Chitosan/hyaluronic acid nanoparticle-mediated delivery	Vascular endothelial cells and atherosclerotic plaques	Improved plaque accumulation and provided a platform for local delivery of anti-inflammatory or lipid-modulating therapeutics	Preclinical platform; therapeutic payload, long-term efficacy, and safety remain uncertain	(81)
Platelet membrane-coated anti-CD47 nanocarrier	aCD47@PMSN	ApoE−/− mouse atherosclerosis model; vascular injury-related targeting model	Platelet membrane-coated mesoporous silica nanoparticles loaded with anti-CD47 antibodies	Vascular injury sites, atherosclerotic lesions, and apoptotic plaque cells	Enhanced lesion homing, promoted macrophage efferocytosis, reduced lesion area, and improved fibrous cap stability	Potential platelet-related effects, manufacturing complexity, and long-term safety remain unresolved	(82, 83)
Dual-functional anti-CD47 nanotherapy	DNPC-αCD47 loaded with CY-09	ApoE−/− mouse atherosclerosis model	Anti-CD47 antibody-modified nanoparticles loaded with NLRP3 inflammasome inhibitor CY-09	Plaque macrophages; CD47–SIRPα and NLRP3 inflammatory pathways	Blocked CD47–SIRPα signaling, inhibited NLRP3-mediated inflammation, reduced IL-1β expression, decreased plaque burden, and reduced necrotic core area	Dual-functional nanosystem remains preclinical; standardization, scalability, and human safety remain unknown	(84)
Large-animal pro-efferocytic nanotherapy	SWNT-based SHP-1 inhibitor delivery	LDLR−/− pig atherosclerosis model	Single-walled carbon nanotube-mediated SHP-1 inhibitor delivery	Inflammatory monocyte–macrophage populations in plaques	Enhanced efferocytosis, reduced apoptotic cell accumulation, and alleviated plaque inflammation without overt anemia or thrombocytopenia	Large-animal evidence improves translational relevance, but dosing, biodistribution, and long-term safety require further study	(79, 85)
CD47 “self-marker” biomimetic nanocarrier	CD47- or CD47-mimetic peptide-modified nanoparticles; cell membrane-coated nanocarriers	Atherosclerosis-related nanodelivery models	Macrophage, erythrocyte, or hybrid membrane coating; CD47-mediated self-camouflage	Reticuloendothelial system evasion and plaque-targeted delivery	Prolonged circulation time, reduced hepatic and splenic clearance, and improved plaque targeting	This strategy is conceptually distinct from therapeutic CD47 blockade; clinical manufacturing and safety remain unresolved	(86–88)

ApoE−/−, apolipoprotein E-deficient; LDLR−/−, low-density lipoprotein receptor-deficient; VSMCs, vascular smooth muscle cells; SIRPα, signal regulatory protein α; SHP-1, Src homology region 2 domain-containing phosphatase-1; CS–HA, chitosan/hyaluronic acid; PMSN, platelet membrane-coated mesoporous silica nanoparticles; DNPC-αCD47, dual-functional nanoparticles modified with anti-CD47 antibody; CY-09, NLRP3 inflammasome inhibitor; IL-10, interleukin-10; SWNTs, single-walled carbon nanotubes. Some interventions are supported by indirect evidence from oncology, vascular injury, metabolic disease, or nanodelivery models and should be interpreted as hypothesis-generating rather than direct clinical evidence for atherosclerosis.

For clarity, CD47-centered therapeutic strategies can be classified into four mechanistic categories. The first category is direct CD47/SIRPα blockade, including anti-CD47 antibodies, SIRPα blockers, and SIRPα–Fc fusion proteins, which aim to release macrophages from inhibitory phagocytic checkpoint signaling. The second category is downstream checkpoint modulation, including SHP-1/2-related small-molecule or RNA-based approaches, which indirectly restore macrophage efferocytosis. The third category is plaque-targeted delivery, which aims to improve lesion selectivity and reduce systemic exposure. The fourth category is CD47-mediated immune camouflage, in which CD47 or CD47-mimetic signals are used to prolong nanoparticle circulation rather than to block CD47 signaling. This classification helps distinguish therapeutic CD47 blockade from delivery-oriented exploitation of CD47 biology.

### 6.1 The biological basis of CD47-targeted therapy

Therapeutically, the most direct rationale for targeting the CD47–SIRPα axis in atherosclerosis is to release macrophages from inhibitory “don’t-eat-me” signaling and restore efferocytosis. In advanced plaques, excessive CD47 signaling on apoptotic cells, foam cells, and VSMC-derived plaque cells suppresses macrophage-mediated clearance, thereby promoting apoptotic cell accumulation, necrotic core expansion, and persistent inflammation. Therefore, CD47–SIRPα blockade may stabilize plaques by enhancing efferocytosis, limiting secondary necrosis, and promoting inflammation resolution.

In parallel, the TSP-1–CD47 axis provides additional therapeutic entry points by regulating endothelial NO–cGMP signaling, oxidative stress, angiogenic responsiveness, macrophage macropinocytosis, platelet activation, and VSMC remodeling. However, because TSP-1–CD47 signaling also participates in physiological vascular homeostasis and tissue repair, therapeutic intervention in this pathway requires greater caution. Thus, CD47-centered therapy in atherosclerosis should not be viewed as simple systemic CD47 inhibition, but rather as context-specific modulation of plaque macrophages, vascular cells, and inflammatory signaling.

### 6.2 Anti-CD47 monoclonal antibody: exploration in the field of oncology

The development of anti-CD47 monoclonal antibodies initially centered on oncology, with Hu5F9-G4, later named magrolimab, being the first representative agent to enter human clinical testing. In a first-in-human phase I dose-escalation study in patients with advanced solid tumors, Hu5F9-G4 employed a priming-dose strategy followed by maintenance dosing to achieve gradual occupancy of CD47 on red blood cells, thereby mitigating acute hemolytic reactions. This study established the clinical feasibility of CD47 blockade and identified its primary toxicities as transient anemia and mild-to-moderate infusion-related reactions (65). Building on these findings, magrolimab rapidly advanced into clinical trials for myelodysplastic syndromes (MDS) and acute myeloid leukemia (AML). In a phase Ib study in high-risk MDS, Sallman et al. reported that magrolimab combined with azacitidine yielded encouraging response signals, including increased complete remission rates in patients harboring TP53 mutations, while maintaining a generally manageable safety profile (66).

However, these early encouraging results were not consistently confirmed in later-stage clinical development. In the ENHANCE-2 trial conducted by Zeidner et al. in TP53-mutant MDS/AML, magrolimab plus azacitidine failed to improve overall survival compared with physician’s choice therapy, and in several subgroups the magrolimab arm exhibited even shorter median survival. As a result, the trial was terminated early at interim analysis due to futility (67). In addition, the phase III ENHANCE study in higher-risk MDS was discontinued because of futility based on a planned analysis. In 2024, the phase III ENHANCE-3 study in AML was also discontinued, and the U.S. Food and Drug Administration placed all magrolimab studies in MDS and AML, including related expanded access programs, on full clinical hold (68). These findings indicate that a strong mechanistic rationale and early-phase response signals do not necessarily translate into durable clinical benefit or acceptable safety in larger randomized trials.

For cardiovascular translation, these setbacks are particularly important. Unlike oncology, atherosclerosis is a chronic disease that would likely require long-term or repeated therapeutic exposure. Patients with atherosclerosis may also have baseline anemia, renal dysfunction, diabetes, antiplatelet or anticoagulant use, and elevated thrombotic or bleeding risk. Therefore, the hematologic toxicity, platelet-related effects, and off-target phagocytosis observed or anticipated during systemic CD47 blockade must be carefully considered before extending anti-CD47 therapy to cardiovascular disease (69, 70).

Drawing from these clinical lessons, CD47-targeting strategies have progressively shifted from first-generation antibodies toward newer molecules engineered to reduce red blood cell binding, hemagglutination, and platelet-related toxicity. For example, IMC-002 structurally reduces its affinity for CD47 on red blood cells, thereby mitigating anemia and other dose-limiting toxicities. In a phase I study in solid tumors, no dose-limiting toxicities were observed at doses up to 30 mg/kg administered every two weeks, indicating a favorable tolerability profile (71). AK117 (ligufalimab) substantially reduces red blood cell agglutination by targeting an alternative CD47 epitope and exhibits lower hematologic toxicity compared with conventional anti-CD47 antibodies (72). Nevertheless, these agents remain primarily oncology-oriented, and their relevance to atherosclerosis is indirect. Whether such designs can achieve sufficient plaque selectivity, long-term safety, and clinically meaningful vascular benefit remains unknown.

### 6.3 The application of anti-CD47 in atherosclerosis

In atherosclerosis, therapeutic strategies targeting the CD47–SIRPα axis are being explored to enhance macrophage efferocytosis, reduce necrotic core formation, limit inflammatory amplification, and promote plaque stabilization. Kojima and colleagues demonstrated across multiple mouse models and human atherosclerotic specimens that CD47 is markedly upregulated within plaques and positively correlates with necrotic core enlargement and adverse cardiovascular risk, providing pathological evidence in support of CD47-targeted therapeutic intervention (11).

Further mechanistic studies indicate that the therapeutic effects of anti-CD47 treatment are not exerted in isolation, but rather depend on the intrinsic capacity of macrophages to clear damaged cells. Mueller and colleagues found that the reduction in plaque burden mediated by anti-CD47 antibodies is closely linked to macrophage low-density lipoprotein receptor-related protein 1 (LRP1) signaling. When LRP1 was deleted in macrophages, the enhanced phagocytic activity induced by CD47 blockade was markedly attenuated, suggesting that LRP1 serves as a critical mediator through which CD47 immunomodulation exerts therapeutic benefit in atherosclerosis (17).

#### 6.3.1 Anti-CD47 antibody and SIRPα blocker

This finding reinforces the importance of functional efferocytosis pathways in plaque stabilization and suggests that CD47-targeted therapy may need to be integrated with the macrophage clearance capacity of the plaque microenvironment (73). These strategies aim to disrupt the CD47–SIRPα interaction and restore macrophage efferocytosis by releasing macrophages from inhibitory “don’t-eat-me” signaling (74). During apoptosis, CD47 redistribution from clustered lipid raft microdomains to a more dispersed membrane pattern reduces its binding avidity to macrophage SIRPα, thereby facilitating apoptotic cell engulfment (75). Based on this mechanism, anti-CD47 antibodies or SIRPα-blocking agents may promote the clearance of apoptotic foam cells, apoptotic VSMC-derived cells, and lipid-rich debris, thereby reducing necrotic core accumulation and improving features of plaque stability. In ApoE−/− mice, anti-CD47 antibody treatment reduced plaque burden, enhanced macrophage efferocytosis, and shifted plaque architecture toward a more stable phenotype characterized by smaller necrotic cores and thicker fibrous caps (17). In vascular injury and restenosis models, CD47 blockade has also demonstrated potential benefits by enhancing phagocytosis and suppressing proliferative responses. In a mouse carotid artery injury model, Govatati et al. showed that anti-CD47 treatment promoted engulfment of apoptotic VSMCs and inhibited their migration and proliferation, thereby reducing restenosis severity (32). However, this evidence should be distinguished from spontaneous atherosclerotic plaque models, because restenosis and plaque vulnerability involve overlapping but not identical biological processes.

From the receptor side, SIRPα is an inhibitory receptor expressed on macrophages whose intracellular ITIM domains mediate negative signaling through recruitment of SHP-1/2. Mice with myeloid cell-specific deletion of SIRPα display reduced plaque burden and attenuated inflammatory responses under high-fat diet conditions, indicating that SIRPα acts as a critical brake on macrophage effector functions during atherosclerosis progression (76). Engineered high-affinity SIRPα–Fc fusion proteins can competitively bind CD47 as soluble receptors and disrupt endogenous CD47–SIRPα interactions. However, most current clinical development of these agents remains in oncology rather than cardiovascular disease (37).

Because CD47 is broadly expressed among diverse cell types, particularly erythrocytes, neutrophils, and platelets, pharmacologic blockade of CD47 signaling may enhance splenic macrophage clearance of these cells, leading to hematologic adverse effects such as anemia, neutropenia, and thrombocytopenia (69, 70). Thus, a key challenge for next-generation drug optimization and delivery strategy design is to achieve high selectivity toward plaque-associated pathological cells without substantially compromising the survival of erythrocytes and platelets.

#### 6.3.2 Small molecule drugs and RNA intervention strategies

Compared with large-molecule antibodies and fusion proteins, small-molecule inhibitors and RNA-based interventions may offer advantages in tissue penetration, intracellular target modulation, routes of administration, and manufacturing cost. Key downstream signaling components of the CD47–SIRPα axis include SHP-1/2 and the guanine nucleotide exchange factor Vav, which regulates Rac family GTPases and phagocytic cytoskeletal remodeling. Mechanistic studies have shown that CD47 inhibits Vav phosphorylation through activation of the SIRPα–SHP-1/2 pathway, thereby suppressing Rac-dependent phagocytic circuits and impairing macrophage phagocytosis (77). Within this context, small-molecule SHP-1 inhibitors have emerged as a potential strategy to release the “don’t-eat-me” inhibitory signal. In atherosclerosis-related studies, either genetic deletion of SHP-1 in myeloid cells or delivery of SHP-1 inhibitors encapsulated in macrophage-targeting nanocarriers enhanced apoptotic cell clearance, reduced inflammatory activation, and limited plaque progression (78, 79).

RNA-based interventions may also modulate CD47-centered signaling therapeutically. Chen et al. reported that miR-378a regulates macrophage phagocytic capacity by modulating SIRPα-mediated signaling within the CD47–SIRPα axis. In ApoE−/− mice, downregulation of miR-378a was associated with reduced macrophage efferocytosis and accelerated plaque progression, whereas administration of a miR-378a mimic enhanced foam cell clearance and decreased inflammatory mediator production (53). Other non-coding RNAs, such as lncRNA MIAT, have likewise been shown to influence the balance between “eat-me” and “don’t-eat-me” signals by regulating CD47 or related pathways. Silencing MIAT promoted macrophage efferocytosis, reduced necrotic core formation, and improved plaque stability (38).

From a therapeutic perspective, RNA-based strategies targeting the CD47–SIRPα axis can be broadly divided into two categories: direct suppression of CD47 expression, such as siRNA or antisense oligonucleotides targeting CD47 mRNA, and modulation of SIRPα-related upstream miRNAs, such as miR-378a, to attenuate SIRPα-mediated inhibitory signaling. Regulation of downstream effectors, such as SHP-1/2 or Vav, may also indirectly relieve CD47–SIRPα-mediated inhibition and restore macrophage effector functions (78, 80). However, RNA-based therapies still face challenges related to delivery efficiency, cell specificity, off-target effects, and long-term cardiovascular safety.

Combination strategies may be particularly attractive because plaque vulnerability involves both defective efferocytosis and unresolved inflammation. For instance, co-delivery of a SHP-1 inhibitor together with the anti-inflammatory cytokine IL-10 via macrophage-targeted nanoparticles simultaneously enhanced phagocytic capacity and promoted inflammation resolution, resulting in reduced plaque burden and improved plaque stability in murine atherosclerosis models (80).

#### 6.3.3 CD47-modified nano-targeted drug delivery system

Because systemic CD47 blockade may cause hematologic toxicity and off-target phagocytosis, plaque-targeted nanodelivery systems have been developed to increase local drug accumulation and reduce systemic exposure. These platforms include anti-CD47 antibody-modified nanoparticles, platelet membrane-coated systems, macrophage- or erythrocyte-membrane biomimetic carriers, and nanocarriers delivering SHP-1 inhibitors, anti-inflammatory agents, or RNA therapeutics. However, these systems remain largely preclinical and face major translational challenges related to manufacturing consistency, scalability, biodistribution, tissue retention, and long-term safety.

Yu et al. developed chitosan/hyaluronic acid-based nanoparticles carrying anti-CD47 antibodies, which showed selective binding to vascular endothelial cells *in vitro* and preferential accumulation within atherosclerotic plaques in ApoE−/− mice (81). Chen et al. further used platelet membrane-coated mesoporous silica nanoparticles loaded with anti-CD47 antibodies to enhance homing to vascular injury and atherosclerotic lesions (82, 83). By blocking CD47 on necrotic core-derived smooth muscle cells and other apoptotic cells, this system enhanced macrophage efferocytosis, reduced lesion area, and improved fibrous cap stability. Nevertheless, these systems remain preclinical, and their long-term safety and translational feasibility remain uncertain.

Luo et al. designed dual-functional nanoparticles modified with anti-CD47 antibodies and loaded with the NLRP3 inflammasome inhibitor CY-09. This system simultaneously blocked the CD47–SIRPα checkpoint and inhibited NLRP3-mediated inflammatory signaling in plaque macrophages, thereby reducing NLRP3 and IL-1β expression, promoting apoptotic cell clearance, and reducing plaque burden and necrotic core area in ApoE−/− mice (84). Whether such dual-functional systems can be safely manufactured, standardized, and translated to humans remains unknown.

Other nanotherapeutic strategies have targeted CD47-related downstream inhibitory signaling. Patel et al. co-loaded the SHP-1 inhibitor NSC87877 and IL-10 into polymeric nanoparticles, which promoted macrophage efferocytosis, upregulated anti-inflammatory mediators, and suppressed pro-inflammatory cytokines, ultimately achieving both plaque burden reduction and inflammation resolution (80). Bamezai et al. employed single-walled carbon nanotubes to deliver a SHP-1 inhibitor to inflammatory monocyte–macrophage populations within atherosclerotic plaques of LDLR−/− pigs. This “pro-efferocytic nanotherapy” reduced apoptotic cell accumulation and local inflammation in a large-animal model without inducing notable anemia (79, 85). The use of a large-animal model strengthens translational relevance, but clinical applicability remains limited by safety, delivery, and manufacturing concerns.

A distinct strategy utilizes CD47 as a “self-marking” molecule. By displaying CD47 or CD47-mimetic peptides on nanoparticle surfaces, nanoparticles can evade reticuloendothelial clearance, prolong circulation time, and increase exposure to diseased vascular walls (86). In atherosclerosis-related nanosystems, macrophage membranes, erythrocyte membranes, or hybrid cell membranes are frequently used to cloak polymeric or inorganic nanocores. The presence of endogenous CD47 on these biomimetic coatings helps reduce hepatic and splenic clearance, while membrane surface receptors, such as PSGL-1 and integrins, facilitate plaque targeting (81–83, 87, 88). However, this “self-camouflage” strategy is conceptually different from therapeutic CD47 blockade and should be clearly distinguished from anti-CD47 approaches.

Overall, CD47-centered therapeutic strategies in atherosclerosis remain at an early translational stage. Antibody and SIRPα-blocking approaches provide direct mechanistic support for restoring efferocytosis, small-molecule and RNA-based strategies offer opportunities for intracellular or post-transcriptional modulation, and nanodelivery systems may improve plaque targeting. Nevertheless, most evidence is derived from animal models, vascular injury models, oncology trials, or indirect mechanistic studies. Therefore, the therapeutic potential of CD47-centered intervention must be evaluated together with its hematologic toxicity, platelet-related risks, off-target effects, and long-term safety, as discussed below.

## 7 Translational limitations and safety concerns of CD47-targeted therapy

Although extensive studies have established the CD47–SIRPα axis as a critical immune checkpoint that restrains macrophage phagocytosis within atherosclerotic plaques, therapeutic agents targeting CD47 have not yet entered clinical trials for atherosclerosis or cardiovascular disease. Clinical translation remains hindered by multiple biological and pharmacological barriers, including systemic hematologic toxicity, off-target phagocytosis, platelet-related adverse effects, uncertain effects on angiogenesis and tissue repair, lack of plaque-specific patient selection tools, limited translational animal evidence, and the immaturity of current delivery technologies.

First, the broad expression of CD47 on erythrocytes, platelets, neutrophils, and other normal cells makes systemic CD47 blockade intrinsically prone to hematologic toxicity. In oncology trials, magrolimab has been associated with hemolytic anemia and thrombocytopenia, necessitating the implementation of a priming-dose strategy to induce erythroid adaptation and mitigate acute hemolysis (89). The high expression of CD47 on erythrocytes normally protects red blood cells from premature macrophage-mediated clearance; therefore, blockade of CD47 can remove this self-protective signal and accelerate erythrocyte consumption (82, 90). Such complex dose-escalation regimens and systemic toxicities pose even greater concerns for patients with atherosclerosis, who frequently present with baseline anemia, impaired renal function, diabetes, or long-term antiplatelet or anticoagulant therapy.

Second, platelet-related adverse effects represent another major translational concern. Under physiological conditions, platelet CD47 engages macrophage SIRPα to deliver a “don’t-eat-me” signal and maintain platelet homeostasis. Systemic blockade of this axis removes this inhibitory safeguard, rendering platelets susceptible to accelerated clearance by the mononuclear phagocyte system and leading to immune thrombocytopenia (91). In addition, many first-generation anti-CD47 antibodies are bivalent IgG molecules that not only bind platelet CD47 with high affinity but also retain an intact Fc domain. As a result, they may promote platelet–platelet crosslinking through Fcγ receptor engagement and/or intrinsic bivalency, leading to platelet aggregation and positive *in vitro* platelet aggregation assays (92). Consistent with this mechanism, the SIRPα-Fc fusion protein TTI-621 has been shown to induce transient thrombocytopenia within hours of administration in a phase I clinical trial, whereas Hu5F9-G4 (magrolimab) reduced platelet counts in animal models and increased mean platelet volume, which has been interpreted as indirect evidence of platelet activation and aggregation (93). Consequently, recent drug design strategies have shifted toward minimizing sustained high-affinity crosslinking of platelet CD47 by employing Fc silencing, IgG4 isotypes, pH-dependent binding, or SIRPα-targeted blockade. These modifications aim to preserve therapeutic activity while reducing platelet aggregation and treatment-associated thrombocytopenia (72).

Third, systemic elimination of the CD47–SIRPα “don’t-eat-me” signal may enhance macrophage phagocytosis in a non-selective manner. Although this effect is beneficial for clearing apoptotic cells and necrotic debris within plaques, it may also promote unintended clearance of normal or stressed cells in non-vascular tissues. Long-term inhibition of CD47 may disrupt tissue homeostasis, senescent cell regulation, infection surveillance, and immune tolerance (38, 94). Other studies have also raised concerns that the ubiquitous expression of CD47 in normal tissues may lead to unintended phagocytic clearance and clinically relevant hematologic toxicities once this inhibitory signal is removed (95). Because atherosclerosis is a chronic disease that would likely require long-term intervention, these potential long-term immune consequences require greater attention than in short-term experimental models or oncology settings.

Beyond quantitative hematologic toxicity, systemic CD47 blockade may exert more complex biological effects through platelet-mediated amplification of inflammation. Recent evidence indicates that activated platelets can release integrin- and tetraspanin-enriched tether-like structures, which enhance platelet adhesion to immune cells and endothelial cells, thereby amplifying both local and systemic inflammatory responses (96). In severe inflammatory models, these platelet-derived tethers promote immune cell recruitment, intensify inflammatory signaling cascades, and exacerbate tissue injury. Given the role of the CD47–SIRPα axis in maintaining platelet immune tolerance and restraining aberrant platelet activation, systemic disruption of this pathway may not only reduce platelet counts but also alter platelet inflammatory phenotypes and functional states. Such changes could further amplify inflammatory responses and increase the risk of tissue damage, which is particularly relevant in atherosclerosis, a chronic inflammatory vascular disease requiring long-term treatment.

Fourth, CD47 signaling is involved in angiogenesis and tissue repair, raising additional safety concerns for systemic CD47-targeted therapies. *In vitro* studies using cerebral endothelial cells have shown that activation of CD47 signaling by thrombospondin-1 suppresses endothelial cell migration and tube formation, both of which are key processes in neovascularization, and increases endothelial cell death (97). Moreover, CD47 has been implicated in age-related declines in vascular function. In aged wild-type mice and human arteries, elevated CD47 and thrombospondin-1 expression correlates with reduced endothelial proliferation, migration, ex vivo sprouting, and *in vivo* angiogenesis, whereas these effects are mitigated in CD47-null models (98). In tumor models, genetic ablation of CD47 in stromal endothelial cells enhances angiogenesis and vascular integrity, together with increased VEGF-A and VEGFR2 expression, further suggesting that CD47 signaling can restrain blood vessel formation and stability *in vivo*(99).

These findings raise important translational considerations. Physiological neovascularization is critical for tissue repair and functional recovery after ischemic injury, such as during the subacute phase after stroke. Systemic inhibition of CD47 may therefore influence post-ischemic revascularization and neurovascular remodeling, potentially affecting recovery processes that depend on endothelial migration and new vessel formation. Although direct clinical data on CD47 blockade in stroke recovery or post-ischemic vascular repair remain limited, the available endothelial biology evidence suggests that unselective global modulation of CD47 signaling warrants cautious assessment in cardiovascular and cerebrovascular disease settings.

Fifth, CD47 expression within atherosclerotic plaques exhibits strong cell-type specificity and dynamic temporal regulation. Evidence indicates that macrophages, smooth muscle cells, and endothelial cells may upregulate CD47 at different pathological stages, with expression levels influenced by inflammatory signaling, cellular stress, and apoptotic status (30). Other studies further indicate that when vascular smooth muscle cells participate in restenosis and vascular repair, functional blockade of CD47 alters their migration and apoptotic responses, suggesting that CD47 expression in this cell population has temporal significance during vascular remodeling (32). However, at present, no clinically validated imaging or circulating biomarkers are available to identify plaques with high CD47 activity or to select patients most likely to benefit from CD47-targeted intervention. This lack of patient stratification tools complicates clinical trial design and increases the risk of exposing low-benefit patients to systemic toxicity.

Sixth, existing animal evidence remains insufficient for direct clinical translation. Although murine studies consistently show that CD47 blockade reduces necrotic core formation and improves features of plaque stability, substantial interspecies differences limit clinical extrapolation. Mice lack spontaneous plaque rupture, display different macrophage subtype distributions, and have markedly shorter lifespans, all of which restrict translational relevance. In contrast, in LDLR−/− pigs, delivery of a SHP-1 inhibitor via single-walled carbon nanotubes improved macrophage efferocytic activity, reduced apoptotic cell accumulation, and alleviated plaque inflammation without inducing overt anemia or thrombocytopenia (79). This large-animal evidence strengthens translational relevance, but further studies are still required to clarify dosing, biodistribution, long-term safety, and durable vascular benefit.

Finally, plaque-targeted nanodelivery systems may reduce systemic exposure but remain far from clinical readiness. To overcome the off-target toxicity caused by systemic administration, multiple research teams have developed plaque-targeted delivery strategies based on nanotechnology, including anti-CD47 nanoparticles encapsulated in platelet membranes (82), multifunctional nanoparticles modified with anti-CD47 antibodies (84), and bio-inspired systems using cell membrane camouflage (83). These strategies substantially improve plaque accumulation, reduce systemic exposure, and enhance local efferocytic and anti-inflammatory activity in murine models. However, from a drug-development perspective, such nanosystems face multiple barriers, including complex fabrication, limited scalability, batch-to-batch variability, uncertain tissue retention, and insufficient long-term safety data. Their chemistry, manufacturing, and quality-control consistency currently remain insufficient for clinical cardiovascular deployment.

Unlike oncology, atherosclerosis is a chronic, non-malignant vascular disease in which therapeutic benefit must be achieved under stricter long-term safety thresholds. Many patients with atherosclerosis require prolonged lipid-lowering, antiplatelet, or anticoagulant therapy, and may have comorbid anemia, renal dysfunction, diabetes, or advanced age. Therefore, future CD47-targeted strategies should be evaluated under conditions that more closely mimic cardiovascular use, including long-term low-dose intervention, localized or plaque-targeted delivery, patient stratification, and concomitant antiplatelet or anticoagulant therapy. Taken together, CD47-targeted therapy for atherosclerosis remains a promising but highly challenging strategy. Future translation will require plaque-selective delivery, reduced erythrocyte and platelet binding, validated biomarkers for patient selection, long-term safety evaluation, and more clinically relevant animal models. Until these issues are resolved, CD47-targeted interventions should be considered preclinical or hypothesis-generating rather than ready for cardiovascular clinical application.

## 8 Future outlook

Future studies should first establish a clearer evidence hierarchy for CD47-centered mechanisms in atherosclerosis. Direct validation in human plaques, advanced atherosclerosis animal models, and large-animal vascular models is needed to distinguish plaque-specific mechanisms from indirect observations derived from oncology, metabolic disease, wound-healing, or vascular injury models. Particular attention should be paid to the cell-type-specific roles of CD47 in macrophages, endothelial cells, VSMCs, platelets, and erythrocytes, because the same molecule may exert distinct effects depending on cellular context and disease stage.

Second, translational research should focus on plaque-selective and safety-oriented therapeutic design. Future CD47-targeted strategies should aim to enhance macrophage efferocytosis within plaques while minimizing erythrocyte binding, platelet clearance, off-target phagocytosis, and systemic immune disturbance. Long-term low-dose regimens, local or plaque-targeted delivery systems, and biomarker-guided patient selection should be prioritized over systemic CD47 blockade. In addition, safety should be evaluated in clinically relevant conditions, including concomitant antiplatelet or anticoagulant therapy, diabetes, renal dysfunction, anemia, and advanced age.

Third, more precise tools are needed to guide clinical translation. Imaging biomarkers, circulating indicators, or plaque molecular signatures reflecting CD47 activity, efferocytosis deficiency, or TSP-1–CD47 vascular stress signaling may help identify patients most likely to benefit from CD47-centered intervention. Ultimately, CD47-targeted therapy for atherosclerosis should be developed not as a simple extension of oncology immunotherapy, but as a cardiovascular-specific strategy that balances plaque stabilization, vascular repair, and long-term systemic safety.

## 9 Conclusion

CD47-centered signaling contributes to both plaque formation and plaque vulnerability through two distinct but interconnected axes. The CD47–SIRPα axis is most strongly supported as an inhibitory phagocytic checkpoint that impairs macrophage efferocytosis and promotes necrotic-core expansion. In parallel, the TSP-1–CD47 axis regulates endothelial dysfunction, oxidative stress, macrophage lipid uptake, vascular smooth muscle cell remodeling, and context-dependent angiogenic responses. Although these pathways provide promising therapeutic targets, most intervention strategies remain preclinical, and their efficacy, cardiovascular safety, and clinical translatability require further validation.
